# A functional polymorphism in the NKG2D gene modulates NK-cell cytotoxicity and is associated with susceptibility to Human Papilloma Virus-related cancers

**DOI:** 10.1038/srep39231

**Published:** 2016-12-20

**Authors:** J. Luis Espinoza, Viet H. Nguyen, Hiroshi Ichimura, Trang T. T. Pham, Cuong H. Nguyen, Thuc V. Pham, Mahmoud I. Elbadry, Katsuji Yoshioka, Junji Tanaka, Ly Q. Trung, Akiyoshi Takami, Shinji Nakao

**Affiliations:** 1 Cellular transplantation Biology, Kanazawa University, Kanazawa, Japan; 2 Department of viral infection and International Health, Graduate School of Medical Sciences, Kanazawa University, Kanazawa, Japan; 3 Hai Phong University of Medicine and Pharmacy, Hai Phong, Vietnam; 4 Division of Molecular Cell Signaling, Cancer Research Institute, Kanazawa University, Kanazawa, Japan; 5 Division of Virology, Department of Laboratory Science, School of Health Sciences, Kanazawa University, Kanazawa, Japan; 6 Soc Trang Provincial Hospital, Soc Trang, Vietnam; 7 Department of Internal Medicine, Division of Hematology, Aichi Medical University, Nagoya, Japan

## Abstract

Human papillomavirus (HPV) is the most common sexually transmitted agent worldwide and is etiologically linked to several cancers, including cervical and genital cancers. NKG2D, an activating receptor expressed by NK cells, plays an important role in cancer immune-surveillance. We analyzed the impact of a NKG2D gene variant, rs1049174, on the incidence of HPV-related cancers in Vietnamese patients and utilized various molecular approaches to elucidate the mechanisms of NKG2D receptor regulation by rs1049174. In a group of 123 patients with HPV+ anogenital cancers, the low cytotoxicity allele LNK was significantly associated with increased cancer susceptibility (p = 0.016). Similar results were also observed in a group of 153 women with cervical cancer (p = 0.05). In functional studies, NK cells from individuals with LNK genotype showed a lower NKG2D expression and displayed less efficient NKG2D-mediated functions than NK cells with HNK genotype. Notably, the rs1049174 variant occurs within a targeting site for miR-1245, a negative regulator of NKG2D expression. Compared with the higher cytotoxicity allele HNK, the LNK allele was more efficiently targeted by miR-1245 and thus determined lower NKG2D expression in NK cells with the LNK genotype. The NKG2D variants may influence cancer immunosurveillance and thus determine susceptibility to various malignancies, including HPV-induced cancers.

Human papillomavirus (HPV) is a double-stranded DNA virus that infects skin and mucosal cells and is the most common sexually transmitted agent worldwide^1^. More than 180 types of HPV have been identified so far, and each type has evolved to infect and propagate in specific epithelial targets, such as the sole of the foot, non-genital skin, anogenital skin, anogenital mucosa and oropharyngeal mucosa^2^. Most HPV infections are subclinical and are typically cleared or suppressed by cell-mediated immunity within 1–2 years of exposure. However, chronic infection and virus persistence commonly occur. Persistent infection with high-risk HPV types may progress to premalignant lesions, and through a multistep process, eventually cause cancers^3^. Infection with the low-risk virus types HPV6 and HPV11 cause nearly 90% of genital warts; conversely, more than 70% of cervical cancers worldwide, and about 50% of cervical intraepithelial neoplasia (CIN) grade 3 (CIN3) are attributed to the 2 most carcinogenic HPV types: HPV16 and HPV18^1^,^2^.

Accounting for an estimated 530,000 new cases and 265,700 deaths in 2012^4^, cervical cancer is the third-most common cancer among women and the second-most frequent cause of cancer-related death worldwide; however, the burden of cervical cancer is disproportionately high, with more than 90% of cervical cancer deaths occurring in developing countries^4^.

Cancer immunosurveillance is based upon the principle that transformed cells naturally rise and are eliminated by the innate immune system before further proliferation^5^. Natural killer (NK) cells are the primary effector lymphocytes of this system and are able to recognize transformed cells without prior education by antigen processing cells^6^. NKG2D, a type II C-type lectin-like family of transmembrane proteins, functions both as an activating and co-stimulatory receptor and is expressed on NK and γδ-cells, as well as subsets of CD4+ and CD8+ T-cells^6^,^7^. NKG2D ligands (NKG2D-Ls), including the MHC class-I chain-related proteins (MICA and MICB) and the UL-16 binding proteins (ULBPs1-4), are almost absent in normal cells but are up-regulated by cell stress events, including cellular transformation or microbial infections. Engagement of the NKG2D receptor with its ligand triggers cell-mediated cytotoxicity and co-stimulates cytokine production even if the target cells have normal HLA class-I expression, promoting the elimination of both infected cells and tumors^6^,^7^.

In an 11-year follow-up study of a general population, Imai *et al*. observed that the overall incidence of cancer was significantly lower in Japanese individuals who had high cytotoxic lymphocytic activity than in those with low cytotoxic activity^8^. Further studies later revealed that this differential in NK cell cytotoxic activity was associated with specific genetic variants mapped to the NKG2D gene. Although those studies did not examine whether or not the difference in cytotoxicity was mediated through the NKG2D receptor, the authors concluded that the high cytotoxicity haplotype HNK was correlated with a lower risk of developing cancer while the LNK haplotype was associated with lower NK cell cytotoxicity and a higher risk of cancer^9^. Due to their high linkage disequilibrium, these haplotypes can be distinguished by the single nucleotide polymorphism (SNP) rs1049174 (+G1163C), located in the 3′-untranslated region (3′UTR) of the NKG2D gene.

We previously demonstrated a significant correlation between the donor HNK genotype and better clinical outcomes in patients with hematological malignancies undergoing hematopoietic stem cell transplantation^10^. Given the involvement of NK cells in immunity against virus-infected cells and their crucial role in the clearance of transformed cells, we hypothesized that NKG2D genotypes determined by the rs1049174 variant may be differentially implicated in the susceptibility to HPV-related cancers.

We therefore determined the frequency of NKG2D genotypes in two cohorts of Vietnamese patients with HPV-related cancers. We also performed various functional studies to characterize the molecular mechanisms by which rs1049174 regulates NKG2D receptor expression in NK cells.

## Design and Methods

### Study population

In this study, DNA samples from 153 cases of cervical cancer and 123 anogenital cancers were obtained from the Vietnam National Cancer Hospital, Hanoi, Vietnam. The diagnosis was based on standard clinical, radiological and histological criteria. DNA samples from 206 randomly selected, age- and ethnicity-matched healthy Vietnamese individuals were used as controls. Control subjects included in this group had no history of cancer or chronic disease. The healthy volunteers gave their informed consent in writing, and the samples from patients were obtained during routine diagnostic procedures after informed consent was obtained. All experiments were performed in accordance with the Declaration of Helsinki and according to institutional guidelines that were approved by the ethics committee of the Hai Phong University of Medicine and Pharmacy in Vietnam and by the ethics committee of Kanazawa University in Japan. The main clinical characteristics of the study population are summarized in Table 1.

### NKG2D Genotyping

NKG2D was genotyped using TaqMan-Allelic discrimination with a StepOne Plus Real Time PCR system (Applied Biosystems, Foster City, CA, USA) as described in a previous report^10^. To avoid confusion, we will refer to the GG genotype as LNK/LNK and the GC and CC genotypes as LNK/HNK and HNK/HNK, respectively.

### Cells lines

The NK cell line YT, generously provided by Dr. K. Kasahara at the Department of Pediatrics of Kanazawa University^11^, and the chronic myeloid leukemia derived cell line OUN-1, kindly provided by Dr. M. Yasukawa from Ehime University in Japan, were cultured in RPMI 1640 + 10% FBS, 100 μg/ml streptomycin and 100 U/ml of penicillin^12^. The primary human lung embryonic fibroblasts (HELs), as previously described^13^, were cultured in DMEM supplemented with 5% newborn calf serum (NBCS) and 2 mM glutamine and used between passages 9–12. HeLa cells were acquired from ATCC (Manassas, VA, USA) and were cultured in DMEM supplemented with 10% FBS.

### Primary NK cell preparation and cell culture

In accordance with the Declaration of Helsinki and according to institutional guidelines and studies that were approved by the ethical committee of Kanazawa University.

Heparinized blood samples were collected from three groups of healthy volunteers. The first group comprised 42 Japanese individuals 20–57 years of age (55% males and 45% females), the second group comprised 33 Japanese individuals 22–24 years of age (35% males and 65% females) and the third group comprised 20 Vietnamese individuals residing in Japan 27–32 years of age (45% male, 55% female). All were unrelated subjects and provided their written informed consent to participate in the study, which was approved by the ethics committee of Kanazawa University. Peripheral blood mononuclear cells (PBMCs) were isolated using the Ficoll-Hypaque gradient (Pharmacia Biotech, Uppsala, Sweden). In some experiments the NK cell fraction was purified using the untouched NK isolation kit (Invitrogen Carlsbad, CA, USA) in accordance with the manufacturer’s instructions. The isolated cells were more than 95% CD3-CD56+CD16+ NK cells, as confirmed by flow cytometry.

### Flow cytometry analysis

Detection of CD3+, CD56+, CD16+, NGK2D+, CD8+or MICA/B+ cells was performed by staining cells with fluorochrome-labeled appropriate antibodies, all from BD Biosciences (San Jose, CA, USA). ULBP ligand detection was performed as described previously^12^. Data acquisition and flow cytometric analyses were performed on a BD FACS Canto Flow cytometer and data was analyzed using the FlowJo v10 software package (Tree Star, Ashland, OR, USA). The NKG2D expression on NK cells from each sample was presented as the mean fluorescence intensity (MFI).

### Infection of cells with human cytomegalovirus (CMV)

The CMV laboratory strain AD169 was used in these experiments. HEL cells were either treated with medium alone or were infected with human CMV at a multiplicity of infection (MOI) of 2. After adsorption of the virus for 1 h at 37 °C, the inoculum was removed, and culture medium was added to the cell cultures. All experiments were performed at Day 4 after infection, and a 90–95% cytopathogenic effect was observed in the cells, which was determined by a microscopic examination. The CMV infection resulted in the expression of the NKG2D ligand ULBP3 on HEL cells, as confirmed by flow cytometry.

### Induction of NKG2D Ligands on Leukemia Cells

To induce the expression of NKG2D-L, the OUN-1 cells were cultured for 24 hours in the presence or absence of valproic acid (VPA), according to a previous report^14^. Effective induction of the MICA/B and ULBP-2 expression on this cell line was determined by flow cytometry.

### Measurement of IFN-γ secretion

Freshly isolated PBMCs cells were suspended in RPMI 1640 + 10% FBS and 50 U/ml IL-2 and cultured for 48 hours (1 × 10^5^/well) in the presence or absence of coated recombinant human MICA/B (R&D systems) as described before^15^. The levels of IFN-γ in the cultured supernatants were determined by an ELISA assay (Mabtech, Nacka Strand, Sweden) following the manufacturer recommendations.

In some experiments PBMCs from healthy individuals with HNK/HNK (n = 3) or LNK/LNK (n = 3) genotypes were cultured for four days in IL-2 (50 IU) containing medium in the presence or the absence of 10 ng/ml of transforming growth factor (TGFβ1) or 5 μg/ml of soluble recombinant MICA (added on first and third days of culture) and the expression of NKG2D receptor on NK cells, defined as CD3− CD56+ lymphocytes, and on CD8+ T cells, defined as CD3+, CD8+ lymphocytes, was assessed by flow cytometry analysis.

### Cytotoxicity assay

VPA-treated or untreated OUN-1 cells and CMV-infected or uninfected HEL cells were used as target cells for NK cell-mediated cytotoxicity, assessed using classical 4-h ^51^Cr release assays as previously described^14^. In these cytotoxicity assays, the effector cells were co-incubated with ^51^Cr-labeled target cells at a 10:1 effector: target ratio (E: T) for OUN-1 cells and 20:1 for HEL cells. These ratios were chosen after preliminary optimization experiments. To block the cytotoxicity by NK cells, the effector cells were then incubated with 5 μg of mouse anti-NKG2D monoclonal antibodies or equal amounts of isotype control antibodies (both from R&D systems) for 30 minutes at 37 °C before incubation with the target cells. The percentage of lysis was determined as described in a previous report^14^.

NK cell cytotoxicity against HeLa cells was determined by flow cytometry. PBMCs from healthy individuals (3 with LNK/LNK and 3 with HNK/HNK) were prepared as above and co-cultured for 4 h at a 10:1 ratio with HeLa cells that had been labeled with CytoTrack Yellow (Biorad, Hercules, CA, USA) in accordance with the manufacturer’s instructions. Co-cultured cells were harvested and stained with anti-CD3, anti-CD56, anti-CD107 and 7AAD. The percentage of CD107+ NK cells (CD3−, CD56+, CD107+) was used as an indicator of degranulation, and the cytotoxicity was assessed as the percentage of 7AAD+ cells in the target population (CytoTrack Yellow+).

### Allelic expression imbalance

Allele-specific transcriptional quantification to determine the potential allelic imbalance between HNK and LNK alleles was carried out basically as described by Hitomi *et al*.^16^, with minor modifications. PBMCs were isolated from three healthy donors with heterozygous NKG2D rs1049174, and genomic DNA and total RNA were isolated. Allelic expression analyses were performed using the TaqMan assay with SNP genotyping probes. The reaction contained the specific probe for rs1049174 genotyping (C_9345347_10). Genomic DNA was used as a control for equal biallelic representation.

### Reporter gene assays

A segment of the 3′ UTR of the NGK2D gene was amplified by polymerase chain reaction (PCR) from the cDNA of two individuals harboring the SNPs “C” or “G”. The 3′ UTRs of the two individuals were nearly identical and differed only in the C or G nucleotide corresponding to the rs1049174 SNP, as confirmed by direct sequencing. PCR was performed with the premiers described previously^11^. These PCR products were then sub-cloned into pGL3 plasmid as described before^11^ to generate pGL3-NKG2D-3′UTR-HNK1 (C) and pGL3-NKG2D-3′UTR-LNK1 (G), designated thereafter as LNK-Luc and HNK-Luc, respectively. The 3′UTRs were inserted with the same orientation relative to each other, and the nucleotide sequences of the 3′UTRs of the plasmids were confirmed by DNA sequencing. Equimolar amounts of each reporter plasmid construct were transfected into HEK293 cells or into YT cells^11^. To control for transfection efficiency, the cells were co-transfected with a renilla reporter plasmid (pRL-TK) with transfection of the different genotypes constructs always running in parallel. The transfected cells were harvested after 48 h, and the activity of both luciferase and renilla were measured using the Dual Luciferase Reporter Assay System (Promega). Luciferase activity was normalized to renilla activity to correct for differences in transfection efficiency. At least three independent experiments were performed for each reporter gene construct in each cell line.

### Prediction of NKG2D 3′ UTR targeting by miR1245

Using the online algorithms TargetScan^17^, miTarget^18^ and MicroSniper^19^, we identified a conserved target site mapping within the rs1049174 SNP in the NKG2D 3′ UTR. HEK cells overexpressing microRNA-1245 were generated as described in a previous report^11^ by lentiviral delivery of a human miR-1245 precursor microRNA overexpression construct (PMIRH1245PA-1-SBI) or a negative control construct vector (pCDH-CMV-MCS-EF1-copGFP) designated hereafter as miR-1245-vector and NC-vector, respectively, following the manufacturer’s recommendations (Systems Biosciences, Mountain View, CA, USA).

### Data analyses

Allele frequencies were calculated with Hardy-Weinberg equilibrium. Differences in the allelic frequencies of the polymorphism between cases and control subjects were compared using a 2 × 2 contingency χ^2^ test or Fisher’s exact test. Student’s *t*-test and the Mann-Whitney *U* test were used to assess the differences in expression between genotypic groups. A *P* value of ≤0.05 was considered statistically significant. The statistical analyses were performed using the GraphPad Prism Software Package (San Diego, CA, USA) and the Microsoft Excel software package, version 2013 (Redmond, WA, USA).

## Results

### Association of NKG2D rs1049174 polymorphism with susceptibility to HPV-related cancer

The characteristics of the studied cases and healthy controls are shown in Table 1. All of the patients studied were positive for HPV. The first group consisted of 153 patients with cervix cancer, most of which were diagnosed as squamous cell carcinoma type (Table 1), and the second group consisted of 123 patients with anogenital cancer, including 49 with penile cancer (39.83%), 49 with vulvar cancer (39.83%), 20 with vaginal cancer (16.26%) and 5 with anal cancer (4.06%). The genotype distributions for the NKG2D polymorphism (rs1049174) among cancer and noncancer subjects are shown in Table 2. The allele frequency for LNK was 0.52, 0.50 and 0.51 in individuals with genital cancer, cervical cancer and overall HPV-cancer, respectively. In contrast, the LNK allele frequency was significantly lower among controls than among cases, 0.42 (p = 0.016). When genotype frequencies were compared among cases and controls, there were no marked differences between the LNK/HNK and LNK/LNK genotypes; however, the frequency of the high cytotoxicity genotype HNK/HNK was significantly lower in patients with genital cancer (p = 0.018) and overall HPV-cancer (p = 0.0158) than in the control individuals.

### Expression of NKG2D in NK cells from HNK genotype individuals

**Figure 1:**
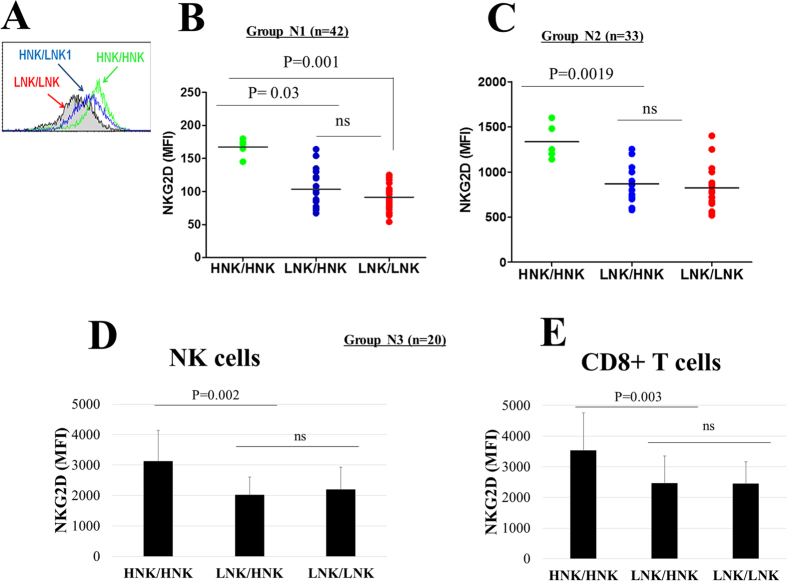
Association between NKG2D genotypes and NKG2D expression on NK cells. (**A**) Representative flow cytometry histogram indicating higher NKG2D expression in NK cells with the HNK/HNK genotype. (**B**) Freshly isolated NK cells from a cohort of 42 healthy Japanese individuals with HNK/HNK (n = 4), LNK/HNK (n = 21) and LNK/LNK (n = 17) genotypes were examined for NKG2D expression at the cell surface by flow cytometry. (**C**) NK samples derived from a cohort of 33 healthy Japanese individuals were assessed for NKG2D expression as in B. The NKG2D expression in NK cells (**D**) and CD8+ T cells (**E**) in a cohort of 20 Vietnamese healthy individuals was assessed by flow cytometry. The MFI values for NKG2D expression are indicated in (**B**,**C**,**D** and **E**) with each dot representing one individual.

Given the location of the rs1049174 SNP in the 3′UTR of NKG2D, we hypothesized that this polymorphism affects NKG2D receptor expression, since NKG2D expression intensity correlates with NK cell-mediated cytotoxicity. To this end, we first assessed NKG2D expression in NK cells derived from a group of 42 healthy Japanese individuals. A flow cytometry examination of freshly isolated NK cells showed higher NKG2D expression in individuals with the minor genotype HNK/HNK than in NK cells isolated from those with the HNK/LNK or LNK/LNK genotype (Fig. 1A and B). Similar results were observed when a more homogenous group (20–24 years of age) of healthy volunteers was examined (Fig. 1C). These findings were further confirmed in a group of 20 Vietnamese individuals. In this group, circulating CD8+ T cells were also examined and NKG2D expression was consistently higher in the NK cells (Fig. 1D) and CD8+ T cells from individuals with the HNK/HNK genotype than in those from individuals with the HNK/LNK or LNK/LNK genotype (Fig. 1E).

### Association of HNK genotypes with the NKG2D-mediated effector function

**Figure 2:**
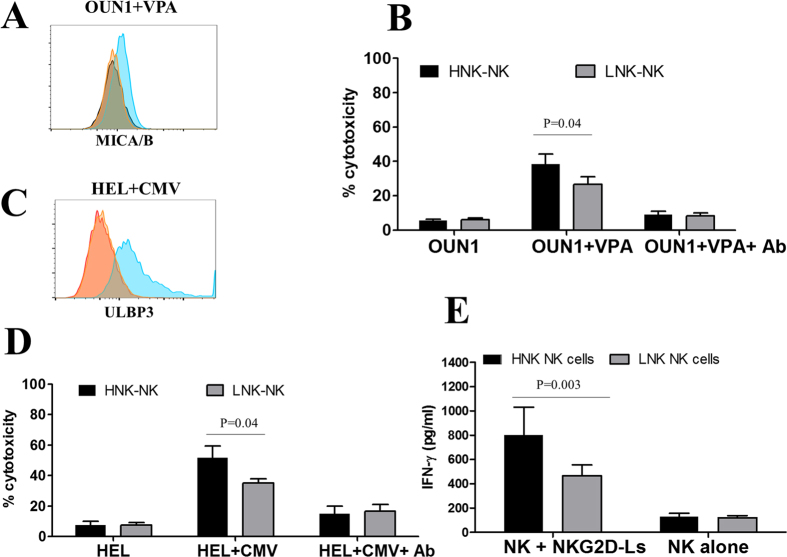
Association between NKG2D genotypes and NKG2D-mediated effector functions. (**A**) The MICA/B expression on the surface of OUN1 cells with (blue histogram) or without VPA treatment (orange histogram), as assessed by flow cytometry, (red histogram; isotype stained cells). The representative results of three independent experiments are shown. (**B**) The cytotoxicity of HNK1/HNK (n = 6) and LNK/LNK (n = 13) NK cells against OUN-1 cells with or without VPA treatment (expressing NKG2D-L) in the presence or absence of a blocking anti-NKG2D mAb, as determined by a ^51^Cr release assay. (**C**) The expression of ULBP3 in uninfected (orange histogram) or CMV infected (blue histogram) HEL cells as assessed by flow cytometry (**D**) Cytotoxicity of HNK1/HNK (n = 6) and LNK/LNK (n = 13) NK cells against CMV-infected human fibroblasts in the presence or absence of a blocking anti-NKG2D mAb. (**E**) IFN-γ secreted by PBMC from individuals with the HNK1/HNK genotype (n = 3) and LNK/LNK (n = 7) cultured with or without NKG2D-Ls and assessed by an ELISA assay.

To determine whether or not the rs1049174 variants correlated with NKG2D-mediated cytotoxicity, NK cells derived from healthy individuals were tested for cytotoxicity against the myeloid leukemia OUN-1 cell line with or without VPA treatment, an inducer of NKG2D-Ls^12^,^14^ (Fig. 2A). Irrespective of the presence of the rs1049174 genotype, treatment of OUN-1 cells with VPA resulted in their increased susceptibility to NK cell cytotoxicity (Fig. 2B). Notably, NK cells derived from individuals with the HNK variant showed higher cytotoxicity against VPA-treated OUN-1 target cells than NK cells from individuals without the HNK variant.

Pre-treatment of leukemic cells with an anti-NKG2D monoclonal antibody abolished the enhanced cytotoxic effect following VPA treatment, and no significant difference in NK-cell cytotoxicity was seen between the HNK and LNK groups (Fig. 2B), suggesting NKG2D-mediated killing as the primary mechanism.

To determine the influence of NKG2D genotype on NK-cell mediated anti-CMV immunosurveillance, CMV-infected fibroblasts were subjected to similar cytotoxicity assays. We chose the CMV AD169 strain, since previous studies have shown that fibroblasts infected with this strain are sensitive to NK cell-mediated cytotoxicity^20^ and constitutively express the NKG2D ligand ULBP3 (Fig. 2C). Regardless of NKG2D genotype, NK cells effectively lysed CMV-infected fibroblasts, but the cytotoxicity by HNK+ NK cells was significantly more efficient than that by LNK+ NK cells. This difference between the HNK and LNK groups was nullified by pre-treating the target cells with an anti-NKG2D monoclonal antibody (Fig. 2D). Consistent with these observations, when PBMCs derived from healthy individuals were stimulated with recombinant NKG2D-Ls *in vitro*, the cells derived from individuals with the HNK genotype secreted higher amounts of IFN-γ than those derived from individuals with the LNK genotype (Fig. 2E). Taken together, these results demonstrate that NK cells harboring the HNK genotype display more efficient NKG2D-dependent immune functions than those with other genotypes.

### Impact of rs1049174 variant on NK-cell cytotoxicity against HPV+ HeLa cells

**Figure 3:**
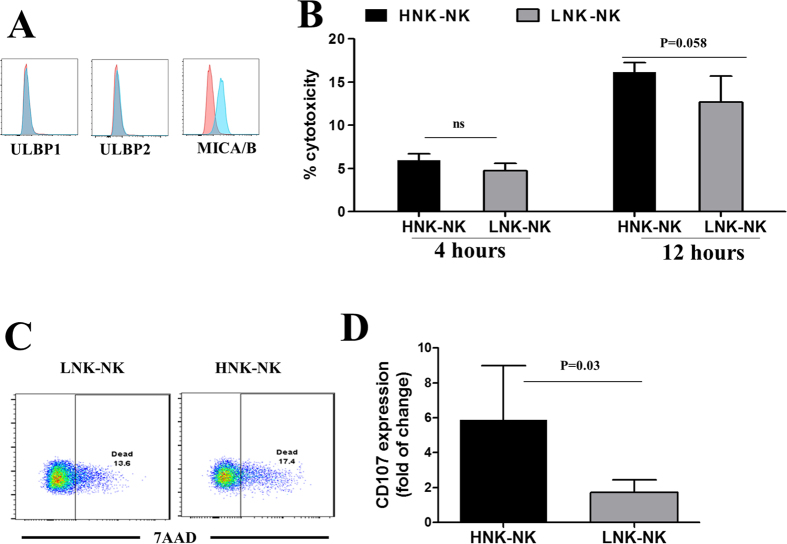
NK cell cytotoxicity against HPV+ cervix cancer cells. (**A**) The constitutive expression of MICA/B but not ULBPs in HeLa cells, as assessed by flow cytometry. The representative results of three independent experiments are shown. (**B**) HeLa cells were labeled with CytoTrack green and then co-cultured with PBMC with HNK1/HNK (n = 3) and LNK/LNK (n = 3) for 4 and 16 h. The frequency of cell death is shown. (**C**) The representative results showing cell death in HeLa cells cultured with PBMCs from an individual with HNK/HNK or LNK/LNK genotype for 16 h, as assessed by flow cytometry. (**D**) NK cells degranulation (CD3−, CD56+, CD107+) after PBMCs from individuals with HNK/HNK (3) or LNK/LNK (3) genotype were co-cultured with HeLa cells for 16 h and assessed by flow cytometry. The data are shown as the standard deviation ± fold-change (% CD107 without target vs. % CD107 with target).

To determine whether or not the rs1049174 variant exerted relevant effects on potential NK-cell cytotoxicity against HPV+ tumor cells, we used a modified flow cytometry approach to simultaneously evaluate target cell lysis and effector cell degranulation. The HeLa cells used as target cells in these experiments were positive for HPV and constitutively expressed high amounts of the NKG2D ligand MICA/B (Fig. 3A). When primary PBMCs were co-cultured with Hela cells for 4 h, little target cell lysis was observed; however, when the co-culture was extended to 16 h, a significant increase in cell cytotoxicity was observed for the NK cells from individuals with the HNK genotype. The cytotoxicity against Hela cells by the LNK cells was significantly lower than that by the HNK cells (Fig. 3B). Consistent with these observations, the HNK NK cells showed three-fold greater degranulation than the LNK NK cells (Fig. 3C), thus substantiating the role of the rs1049174 polymorphism in the modulation of NKG2D-mediated cytotoxicity.

### Molecular characterization of rs1049174 variant

**Figure 4:**
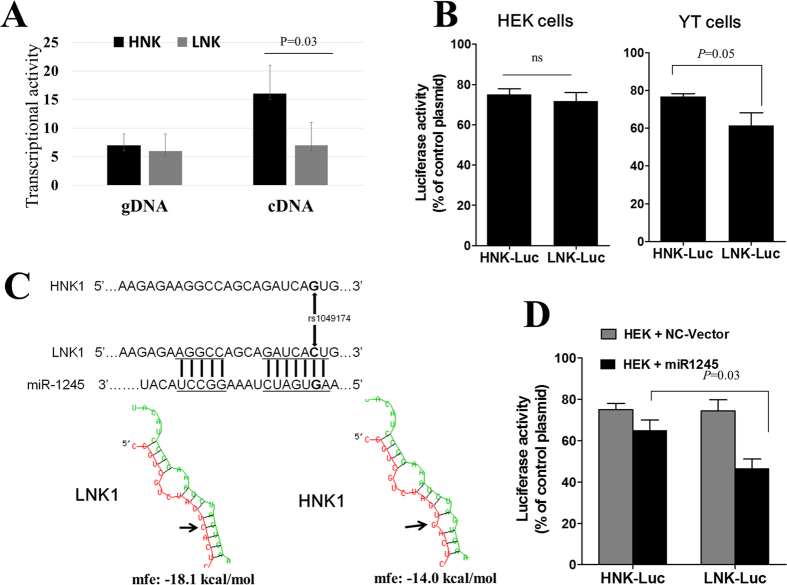
Molecular characterization of rs1049174 polymorphism in the 3′UTR of the NKG2D gene. (**A**) Allele-specific transcriptional activity showing allelic imbalance. The HNK allele had higher transcriptional activity than the LNK allele in cDNA samples but not in gDNA samples from individuals heterozygous for the rs1049174 polymorphism. (**B**) Modulation of reporter gene expression by NKG2D 3′UTR polymorphism. YT cells and HEK cells were transfected with a luciferase expression vector pGL3 TK, or with constructs including the NKG2D 3′UTR HNK1-allele (pGL3 TK HNK1) or the LNK1-allele (pGL3 TK LNK1). The insertion of NKG2D 3′UTR segments repressed luciferase activity. The LNK allele construct, however, repressed the luciferase activity to a significantly greater degree in the YT transfected cells than in the miR1245-lacking HEK cells. (**C**) The rs1049174 SNP 3′UTR region of NKG2D occurs in the miR-1245 binding site, and miR-1245 is expressed in NK cells. A schematic representation of the location 1049174 SNP within the miR-1245 target site in the 3′UTR region of NKG2D. The presence of the HNK genotype (1163C) interrupts the pair-complementarity, decreasing the affinity between NKG2D mRNA and miR-1245. (**D**) The ectopic induction of miR1245 expression in HEK cells resulted in greater luciferase activity repression induced by the LNK allele. HEK293 cells were co-transfected with miR-1245 and pGL3 TK-Luc HNK or pGL3 TK-Luc vectors. At 48 h after transfection, the luciferase activity was measured. Firefly luciferase activity was normalized to renilla luciferase expression, and the means ± the standard error of the percentage of activities in the control situation (cells transfected with luciferase plasmid without NKG2D 3′UTR) are shown.

To determine whether or not the rs1049174 SNP was molecularly involved in the regulation of NKG2D expression, we first performed an Allele-specific transcriptional assay to determine whether or not the rs1049174 variant was associated with allelic imbalance. As shown in Fig. 4A, while no difference in the fluorescence intensity was observed between the HNK and the LNK alleles when genomic DNA samples from individuals heterozygous for the rs1049174 polymorphism were assessed, a 1.9-fold higher fluorescence intensity was observed when cDNA samples from the same heterozygous individuals were tested, thus indicating the existence of an allelic imbalance, with the HNK allele showing higher transcriptional activity than the LNK allele.

To further characterize the rs1049174 variant, we next utilized a luciferase reporter assay. Fragments of the 3′UTR region of the NKG2D gene that included the rs10491474 SNP (HNK allele or LNK allele) were fused to the luciferase open reading frame, and the resultant constructs (HNK-Luc and LNK-Luc) were then transfected into the NK cell line YT or into HEK293 cells. Irrespective of genotypes, the luciferase activity was repressed by constructs containing the 3′UTR of NKG2D. However, significantly higher repression was induced by the construct containing the LNK allele (LNK-Luc) and the difference in the repressive effects between the 2 alleles was less evident in the HEK293 cells in comparison with that induced in YT cells (Fig. 4B). These results suggest that the fragment of the 3′UTR region of the NKG2D gene where the rs1049174 SNP occurs may contain target sites for repressors of NKG2D expression, with the LNK allele possibly possessing greater affinity for potential negative regulators.

To identify specific regulators of NKG2D expression we focused on micro-RNAs, a class of small non-coding RNAs that target specific sites in the 3′ UTR of mRNA that suppress gene expression through translation inhibition or mRNA degradation. Using three different online algorithms (TargetScan, miTarget and MicroSniper) we found that the rs1049174 SNP in the NKG2D 3′UTR resides in a conserved region which represents a possible targeting site for miR-1245, a microRNA that was previously reported to downregulate NKG2D expression in NK cells^11^. Interestingly, the HNK allele interrupts the interaction with the miRNA complementary seed, impairing miR-1245 targeting (Fig. 4C). To directly test whether or not the rs1049174 SNP in the NKG2D 3′UTR was targeted by miR-1245, we performed luciferase assays in the presence or absence of ectopic miR-1245 (LNK-Luc + miR-1245 or HNK-Luc + miR-1245). HEK293 cells, which express low levels of endogenous miR-1245^11^, were co-transfected with miR-1245 and the LNK-Luc construct and showed a 54% decline in luciferase activity compared to LNK-Luc-only controls. Co-transfection of miR-1245 with HNK-Luc constructs, though, resulted in only a 17% decline in luciferase activity compared with controls (Fig. 4D). Taken together, these results indicate that the miR-1245 targets the NKG2D 3′UTR and that the targeting occurs with higher affinity for the LNK allele than the HNK allele.

### Effects of rs1049174 variant on NK cells susceptibility to TGFβ1

**Figure 5:**
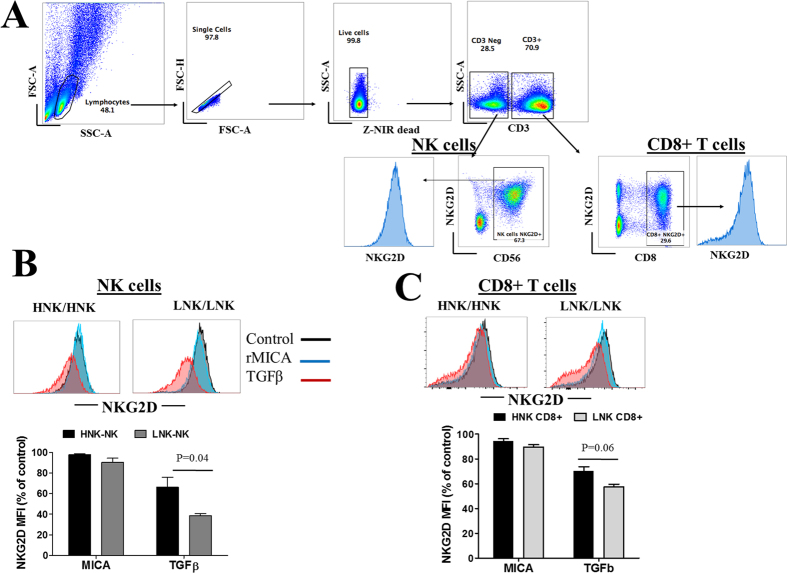
Effects of rs1049174 variant on NK cells susceptibility to transforming growth factor. PBMCs from individuals with HNK/HNK (3) or LNK/LNK (3) genotype were cultured for four days in IL-2 containing medium in the presence of either recombinant MICA (NKG2D-L) or TGFβ1 (10 ng/ml) and the expression of NKG2D receptor on NK cells and CD8+ T cells was assessed by flow cytometry. (**A**) The gating strategy used to assess NKG2D expression in NK cells and CD8+ T cells. (**B**) A representative histogram figure showing NKG2D expression in control (dark histogram), rMICA treated, (blue histogram) or TGFβ1 treated (red histogram) NK cells is shown in the upper panel. The bottom panel shows summarized results from different individuals with HNK1/HNK (n = 3) and LNK/LNK (n = 3) and the data represent the NKG2D intensity (% MFI) in treated cells compared with that in the control cells. (**C**) A representative histogram figure showing NKG2D expression in control (dark histogram), rMICA treated, (blue histogram) or TGFβ1 treated (red histogram) CD8+ T cells is shown in the upper panel. The bottom panel shows summarized results from different individuals with HNK1/HNK (n = 3) and LNK/LNK (n = 3) and the data represent the NKG2D intensity (% MFI) in treated cells compared with that in the control cells.

Transforming growth factor (TGF-β) is a soluble immunosuppressive cytokine that impairs NK cells cytotoxicity, largely by downregulating NKG2D receptor on NK cells^7^. To investigate whether the rs1049174 variant influence the susceptibility of NKG2D expressing cells to the suppressive effects of TGF-β, we cultured PBMCs from individuals with the HNK/HNK and LNK/LNK genotypes in the presence of TGF-β1 and then determined the NKG2D expression on NK cells and CD8+ T cells (Fig. 5A). Irrespective of genotypes, the NKG2D expression on the surface of NK cells was substantially downregulated after cell treatment with TGF-β1. However, compared with NK cells with the HNK/HNK genotype, significantly higher NKG2D suppression was induced in NK cells possessing the LNK/LNK genotype (Fig. 5B). In addition, CD8+ T cells with the LNK/LNK genotype were also more susceptible to the suppressive effects TGF-β1 on NKG2D expression, although the effect was not statistical significant (Fig. 5C). In the same set of experiments we also treated PBMCs with recombinant MICA, since sustained stimulation with NKG2D ligands has been reported to downregulate NKG2D receptor in NK cells^7^, however under these experimental conditions the downregulation of NKG2D receptor in NK and CD8+ T cells induced by MICA stimulation was minimal and no difference was observed between cells possessing the HNK and LNK genotypes (Fig. 5B and C).

## Discussion

This study revealed a higher frequency of the LNK allele of NKG2D gene in Vietnamese individuals with HPV-related cancers than in healthy individuals. We also showed that the rs1049174 polymorphism modulates NKG2D expression and NKG2D-mediated cytotoxicity in both Japanese and Vietnamese individuals. Previous studies have linked the HNK genotype with a lower incidence of colorectal cancer^9^,^21^, and conflicting data were reported in individuals with head and neck and esophagus cancer, where the risk of these cancers in heavy drinkers and smokers was actually higher in those possessing the HNK genotype^22^.

To our knowledge, this is the first study to comprehensively investigate NKG2D receptor regulation by the rs1049174 polymorphism and to demonstrate the effects of this genetic variant in modulating NKG2D-dependent cytotoxicity. At the molecular level, this study demonstrated that the HNK and LNK genotypes resulting from rs1049174 determine the differential sensitivity of the NKG2D gene to a microRNA that negatively regulates NKG2D expression. The microRNA miR-1245 interacts with the 3′UTR region of NKG2D at a binding site that maps within the rs1049174 SNP. The presence of the HNK allele likely interrupts base-pairing complementarity with microRNA-1245, resulting in higher NKG2D expression in NK cells with the HNK allele than in NK cells possessing the LNK allele; as a result, NK cells from individuals with the HNK/HNK genotype had higher levels of NKG2D expression and enhanced cytotoxicity against target cells expressing NKG2D-Ls *in vitro*.

Micro RNAs are single non-coding RNA strands of approximately 22 nucleotides that mediate vital gene regulatory events by pairing with the mRNA of protein coding genes^23^. This interaction leads to the repression of regulatory targets and reduces downstream translational efficiency and mRNA levels^23^. Importantly, the miRNA binding sites are most prevalent in the 3′UTR of mRNA, and therefore, genetic polymorphisms that occur in miRNA target sites can alter miRNA target interactions by disrupting existing binding sites or by creating new miRNA target sites which may alter gene expression^24^. The presence of an SNP in an existing miRNA targeting site may disrupt the binding of miRNA to the target mRNA, and in contrast, an SNP may create a new miRNA binding site. In either case, the final effect of this genetic variation will alter gene expression. Consistent with the data presented here, previous epidemiological and experimental studies have demonstrated that miRNA-related SNPs can affect phenotype and disease risk^24^,^25^.

In a recent meta-analysis that included studies from 5 continents, comprising more than 1 million women with normal cytological findings, the estimated global HPV prevalence was 11.7%. Although the prevalence of HPV infection was highly variable across regions, the most consistently HPV types identified in the analyzed studies were 16, 18, 31, 52, and 58^26^. In a recent cross-sectional study, HPV DNA was detected in 25% of male Vietnamese patients with symptoms related to sexually transmitted infections. HPV52 was the most prevalent high-risk HPV genotype, whereas HPV16 was less common in this study^27^.

In HPV-induced cancers, especially in cervical cancer, screening procedures have become one of the most successful programs for reducing the cancer burden. For example, in most technologically advanced countries the implementation of screening programs have resulted in a nearly 70% decrease in cervical cancer rates over the past 40 years^28^, indicating that prevention is a valuable strategy for reducing the economic and disease burden of HPV infection. HPV-related cancer prevention programs will further benefit from the approval and broad application of two successful prophylactic HPV vaccines—quadrivalent (HPV16/18/6/11) ‘Gardasil’ and bivalent (HPV16/18) ‘Cervarix’—for vaccinating young adolescent girls at or before the onset of puberty^29^, which, as supported by recently published data, prevents the development of cervical lesions in young women, particularly those who have not been infected with vaccine-specific HPV types^29^,^30^.

In this study we focused on the *in vitro* cytotoxicity of NK cells, since most NK cells express the NKG2D receptor and because *in vitro* killing assay involving NK cells are accurate and readily available^31^. However, since several subsets of T cells express varying degrees of NKG2D and are capable of recognizing NKG2D-Ls-expressing target cells, the clinical results presented here may also partially reflect the effect of NKG2D in other cell types, including CD8+ T cells.

Previous studies have shown that, in patients with HPV+ cancers, the CD3+CD8+ infiltration in tumor tissues is associated with increased survival and a good prognosis^32^,^33^, suggesting that a strong interaction between the immune system and tumor cells exists and that an effective cytotoxic cell immune response may be capable of clearing HPV-transformed cells, particularly in non-advanced stages. As described above, acquired immune responses are primarily responsible for clearing HPV infection, and an efficient T cell response is required for this purpose, as immunodeficient patients, such as those with HIV, frequently develop HPV infection reactivation^34^. In addition to acquired immune responses, innate immune responses, including mucosal defense and particularly NK cells, have an active and important role^35^. A potential role of NK cells in eliminating HPV-transformed cells is supported by the observations that NKG2D-Ls expression, including ULBP1, MICA/B and ULBP2, in cervical cancer specimens was found to be an indicator of good prognosis in patients with cervical cancer, suggesting that ligand-expressing cells can be targeted by NKG2D receptor-expressing immune cells *in vivo*^36^. In addition, cervical cancer cell lines express NKG2D-Ls and interact with NKG2D-expressing NK cells *in vitro*^37^.

Various epidemiological studies have attempted to identify genetic factors that differentially influence the risk of HPV-related malignancies in diverse populations. Relevant genetic factors identified include the human leucocyte antigen DRB1*1301, which appears to keep women from developing cervical cancer^38^,^39^. In addition, in a large cohort of Japanese patients, the DRB1*1302 allele showed a protective effect against progression from CIN1 to CIN 2/3^40^.

The role of select KIR genes in association with recurrent respiratory papillomatosis (RRP), a rare disease of the larynx and upper airway caused by HPV-6/11, was also recently investigated. In this study, patients lacking activating KIR genes 3DS1 and 2DS1 were more likely to develop a more severe form of RRP than those with the genes^41^, indicating that NK cells may be necessary to trigger an effective immune response against HPV-infected targets.

The higher susceptibility of NK cells with the LNK/LNK genotype to the down regulatory effects of TGFβ1 on NKG2D receptor expression observed in the current study may be due to the fact that TGF-β1 promotes the maturation of endogenous miR-1245 in human NK cells^11^, in a similar fashion as it modulates miR-21 biogenesis in human vascular smooth muscle cells^42^. This phenomenon occurs at the post-transcriptional level and involves the activation of DROSHA microprocessor complex which facilitate the processing of primary transcript into pre-microRNA^42^. This finding is relevant since TGFβ1 promotes the downregulation of activating receptors in NK cells including NKp30 and NKG2D and elevated serum levels of TGFβ1 have been identified in cancer patients^7^. In addition, high TGFβ1 signal expression has been reported in HPV-related malignant tissues, particularly during the early stages of transformation^43^,^44^, which together with the fact that NKG2D receptor is significantly downregulated in patients with HPV-related cancer^45^, suggest that TGFβ1 signal may represent an important mechanism by which HPV-induced tumors evade NKG2D mediated cytotoxicity.

A potential effect of aging on the expression of NKG2D receptor in immune cells has been investigated by various groups and reported data indicate that, whereas there are no age-related changes in the NKG2D expression levels in NK cells^46^, higher frequencies of NKG2D+ CD4+ T cells are detectable in elder individuals, compared with that in young subjects, indicating that NKG2D+ CD4+ T cells constitute a subset of highly differentiated T cells that represent the senescence of the immune system^47^. In the current study the expression of NKG2D receptor in the CD4+ lymphocytes was not assessed, therefore it remains unknown if the rs1049174 variant may influence the NKG2D expression in CD4+ T cells in the aging population.

In conclusion, we identified a functional SNP in the NKG2D gene that is involved in the regulation of NKG2D expression. Studying the association of this polymorphism in individuals with HPV-induced cancer confirms that the HNK/HNK genotype is associated with increased NKG2D-mediated cytotoxicity and reduced susceptibility to cancer. Our findings also suggest a possible mechanism for the superiority of the HNK/HNK genotype and fortify the larger body of evidence regarding the influence of NKG2D on tumor immune surveillance and cancer risk^38^,^39^,^40^,^41^.

## Additional Information

**How to cite this article**: Espinoza, J. L. *et al*. A functional polymorphism in the NKG2D gene modulates NK-cell cytotoxicity and is associated with susceptibility to Human Papilloma Virus-related cancers. *Sci. Rep.* **6**, 39231; doi: 10.1038/srep39231 (2016).

**Publisher's note:** Springer Nature remains neutral with regard to jurisdictional claims in published maps and institutional affiliations.

## Figures and Tables

**Table 1:** Study population characteristics.

	Control	HPV-Cancer
Age (mean ± SD)	45.27 ± 15.36	53.24 ± 12.46
Sex		
Male	41 (20.30%)	53 (19.2%)
Female	165 (79.70%)	223 (80.8%)
Total	206	276
Type of cancer	**Number (%)**	**Age (mean ± SD)**
Anal cancer	5 (1.81%)	66.8 ± 8.58
Penile cancer	49 (17.75%)	55.12 ± 12.07
Vulvar cancer	49 (17.75%)	56.51 ± 12.84
Vaginal cancer	20 (7.25%)	61.6 ± 13.14
Cervical cancer	153 (55.44%)	50.06 ± 11.38

**Table 2:** NKG2D genotype in cancer patients and control.

Genotype	Control	Genital cancer	P	Cervical cancer	P
HNK/HNK	73 (35.43%)	28 (22.76%)	0.001	42 (27.45%)	0.05
LNK/LNK	42 (20.38%)	33 (26.83%)	0.2	43 (28.10%)	0.3
LNK/HNK	91 (44.17%)	62 (50.41%)	0.1	68 (44.45%)	0.2
	206	123		153	
